# Metabolic, transcriptomic, and genetic analyses of candidate genes for seed size in watermelon

**DOI:** 10.3389/fpls.2024.1394724

**Published:** 2024-07-16

**Authors:** Xiqing Wang, Wen Yan, Núria Real, Yunhe Jia, Yongkai Fu, Xuejun Zhang, Haibo You, Yi Cai, Bin Liu

**Affiliations:** ^1^ Horticultural Branch of Heilongjiang Academy of Agricultural Sciences, Harbin, China; ^2^ Center for Research in Vegetable Engineering Technology of Heilongjiang, Harbin, China; ^3^ Plant Pathology, IRTA Cabrils, Cabrils, Spain; ^4^ Hainan Sanya Crops Breeding Trial Center of Xinjiang Academy Agricultural Sciences, Sanya, China; ^5^ Hami-Melon Research Center, Xinjiang Academy of Agricultural Sciences, Urumqi, China

**Keywords:** watermelon, seed size, genetic mapping, transcriptomic analysis, metabolic analysis

## Abstract

Seed size (SS) constitutes a pivotal trait in watermelon breeding. In this study, we present findings from an examination of two watermelon accessions, namely, BW85 and F211. Seeds from BW85 exhibited a significant enlargement compared to those of F211 at 13 days after pollination (DAP), with the maximal disparity in seed length and width manifesting at 17 DAP. A comprehensive study involving both metabolic and transcriptomic analyses indicated a significant enrichment of the ubiquinone and other terpenoid-quinone biosynthesis KEGG pathways. To detect the genetic region governing seed size, a BSA-seq analysis was conducted utilizing the F_2_ (BW85 × F211) population, which resulted in the identification of two adjacent QTLs, namely, *SS6.1* and *SS6.2*, located on chromosomes 6. *SS6.1* spanned from Chr06:4847169 to Chr06:5163486, encompassing 33 genes, while *SS6.2* ranged from Chr06:5379337 to Chr06:5419136, which included only one gene. Among these genes, 11 exhibited a significant differential expression between BW85 and F211 according to transcriptomic analysis. Notably, three genes (*Cla97C06G113960*, *Cla97C06G114180*, and *Cla97C06G114000*) presented a differential expression at both 13 and 17 DAP. Through annotation, *Cla97C06G113960* was identified as a ubiquitin-conjugating enzyme E2, playing a role in the ubiquitin pathway that mediates seed size control. Taken together, our results provide a novel candidate gene influencing the seed size in watermelon, shedding light on the mechanism underlying seed development.

## 1 Introduction

Seeds, as a unique organ in plants, play a pivotal role in their life cycle. Seed size and weight are agronomically important traits that not only influence plant adaptation to environmental stress but also impact overall yield and quality (Wang et al., 2019; Guo et al., 2020; Miao et al., 2020). Seed development involves the regulation of numerous genes responding to developmental and environmental signals (Figueiredo and Köhler, 2014; Li and Li, 2016). Recent studies have revealed some key genes and regulatory pathways controlling plant seed size (Li and Li, 2016; Li et al., 2019), including G protein signal transduction, the ubiquitin–proteasome pathway, mitogen activation protein kinase (MAPK) signaling, several transcription factors, and the perception and homeostasis of plant hormones (Guo et al., 2020).

G protein signal transduction affects multiple physiological activities during plant growth and development. Functional G proteins are heterotrimeric complexes composed of three subunits, Gα, Gβ, and Gγ. In *Arabidopsis*, a Gγ (AGG3)-deficient mutant presents reduced seed size (Chakravorty et al., 2011; Li et al., 2012). The regulation of grain length by these Gγs is mainly dependent on Gα (RGA1), RGB1, and the transcription factor MADS1 (Liu et al., 2018; Sun et al., 2018).

The ubiquitin receptor realization plays a crucial role in seed growth regulation; several genes of the ubiquitin–proteasome system were reported to regulate seed size, such as *DA1*, *DA2*, *DAR1*, and *GW2* (Xiong et al., 2023)—for instance, in *Arabidopsis*, the ubiquitin receptor DA1 together with the E3 ubiquitin ligases DA2 and BB/EOD1 regulates seed growth (Xia et al., 2013). *AtDAR1* is a homologous gene of *DA1*; the seed and organ size, respectively, were increased in DA1 and DAR1 double-mutant (Dong et al., 2017). This collaborative mechanism is mirrored in the DA1 homologue (GW2) in wheat and maize, which has also been implicated in determining kernel size (Li et al., 2010; Bednarek et al., 2012). In *Setaria italica*, a RING-type E3 ligase SGD1 and its E2 partner SiUBC32 are involved in regulating grain weight, grain size, and panicle size (Tang et al., 2023).

The plant mitogen activator polypeptide (MAPK) cascade, consisting of MAPK, MAPK split (MAPKK) and MAPKK split (MAPKKK), plays an important role in various signal transductions. In *A. thaliana*, the *mkk4/mkk5* double-mutant exhibits shortened seeds (Zhang et al., 2017). This cascade’s importance extends to rice, where OsMKKK10, OsMKK4, and OsMAPK6 positively control grain size (Xu et al., 2018). Additionally, regulatory factors such as regulators, regulatory coactivators, and chromatin regulators have also been investigated. Members of the SQUAMOSA promoter binding regulator family, OsSPL13 and OsSPL16, positively impact rice grain size through the regulation of SRS5 and GL7 expression, respectively (Wang et al., 2015; Segami et al., 2017). Similarly, OsmiR396, OsGRF4, and OsGIF have been identified as positive regulators of rice grain size (Li and Li, 2016). In *Arabidopsis*, the B3 domain tracker factors NGAL and KLU effectively regulate seed size (Zhang et al., 2017).

Watermelon exhibits significant variability in seed size, with seed length (SL) ranging from 4.2 to 16.8 mm (Kim et al., 2009). Watermelon seeds are classified into six representative types based on size: giant, big, medium, small, tiny, and tomato seed (Kim et al., 2009). Watermelons designated for seed consumption typically feature larger seeds, while those intended for flesh consumption necessitate relatively smaller seeds (Gong et al., 2022). Therefore, seed size emerges as a crucial agronomic trait in watermelon breeding. Pioneering work by Poole et al. (Porter, 1937) identified small seeds as dominant to medium-sized ones, with the seeds’ length controlled by *l* and *s* genes. Subsequent studies pinpointed four major effects: stable QTLs (ClSS2.2, ClSS6.1, ClSS6.2, and ClSS8.2) governing seed size and weight (Prothro et al., 2012; Meru and McGregor, 2013), affirming the quantitative nature of seed size variation in watermelon (Prothro et al., 2012; Hui-Wen et al., 2016; Gong et al., 2022). Utilizing the CRISPR/Cas9 system, Wang et al. (2021) generated watermelon *Clbg1* mutants, revealing that reduced ABA levels in the *clb1* mutant led to diminished seed size and weight due to a decrease in cell number.

In this study, we employed a BSA-Seq strategy to map the candidate genes responsible for seed size in watermelon, contributing to the enrichment of genetic knowledge regarding seed size control.

## 2 Results

### 2.1 Seeds from BW85 are bigger than those from F211

**Figure 1:**
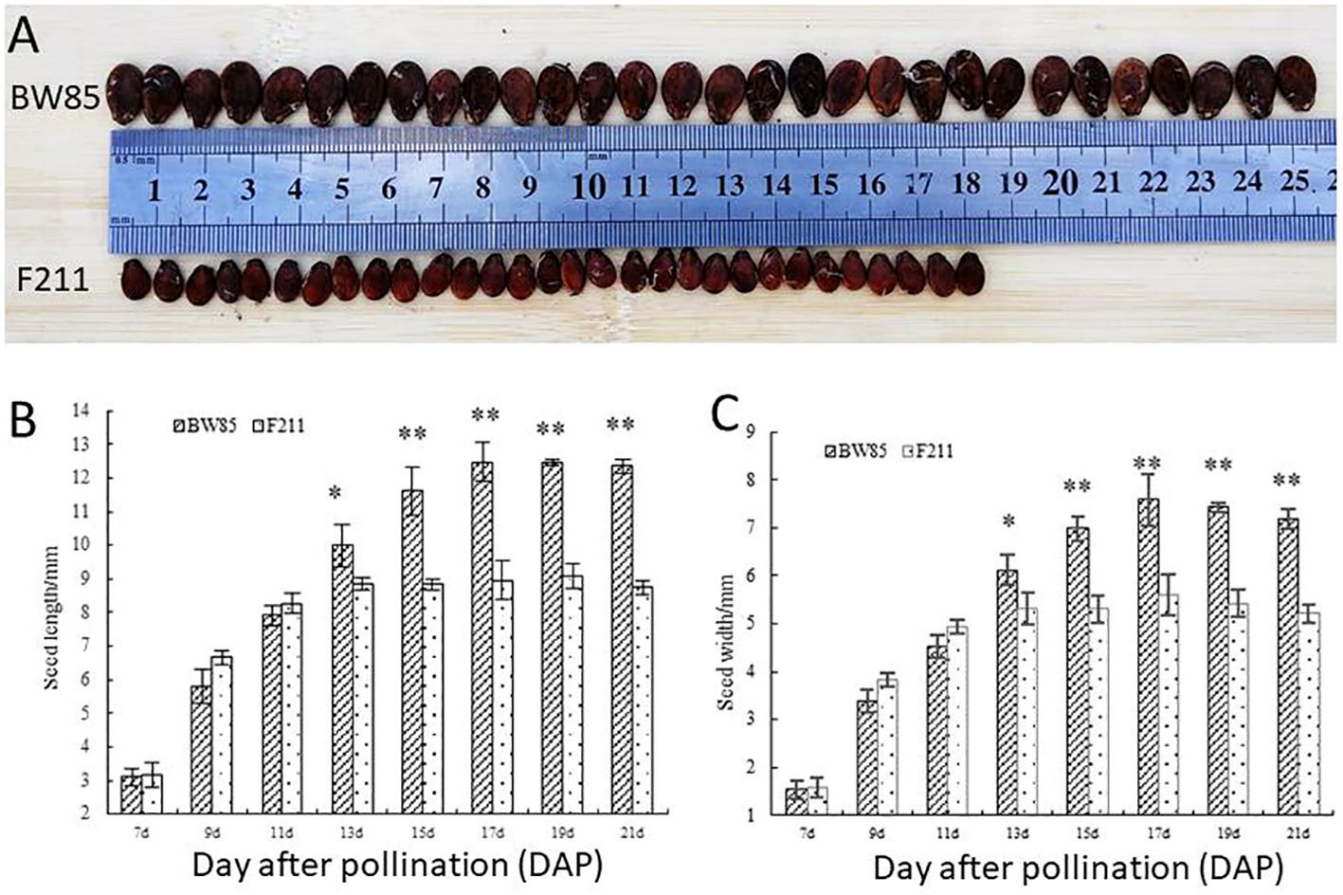
Phenotypic analysis of seed size (SS) of BW85 and F211. **(A)** Seed size of BW85 and F211 at 21 days after pollination (DAP). **(B)** Seed length of BW85 and F211. Error bars represent the standard deviations (SDs; *n* = 20). Asterisks indicate significant differences in seed length between BW85 and F211 at different DAPs (*P* < 0.05). **(C)** Seed width of BW85 and F211. Error bars indicate the standard deviations (SDs; *n* = 20). Asterisks indicate significant differences in seed width between BW85 and F211 at different DAP (*P* < 0.05).

As shown in **Figure 1A**, a clear disparity in seed size between watermelon BW85 and F211 was evident at 21 days after pollination (DAP). Seeds from BW85 exhibited a significant increase in size compared to those from F211 at 13 DAP, with the most pronounced difference in seed length and width observed at 17 DAP (**Figures 1B, C**).

### 2.2 Ubiquinone biosynthesis compounds are different between BW85 and F211 seeds

**Figure 2:**
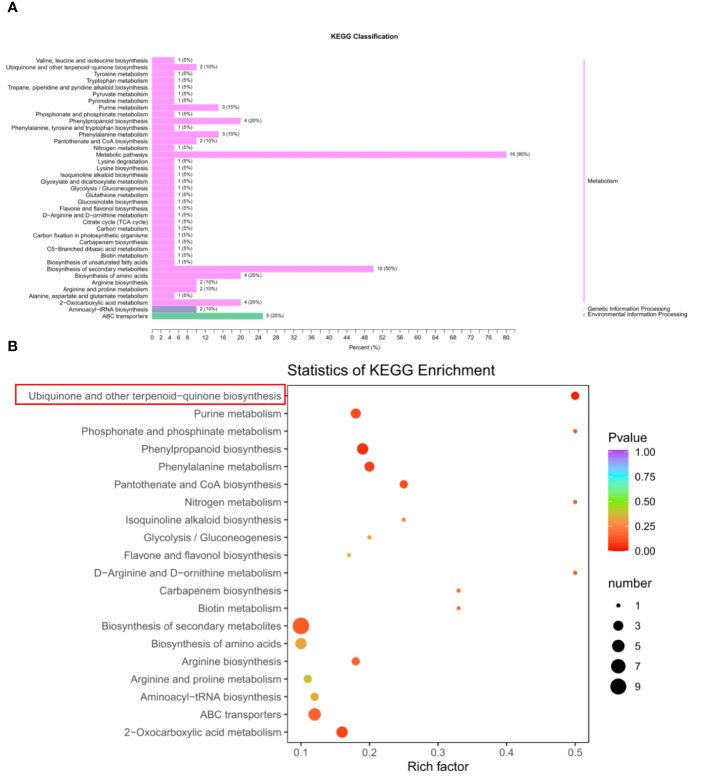
Metabolic analysis of seed development between BW85 and F211 at 13 days after pollination (DAP). **(A)** KEGG classification analysis of different metabolic compounds between BW85 and F211. **(B)** Statistics of KEGG enrichment analysis of different metabolic compounds between BW85 and F211.

To investigate the metabolic differences of seed development between BW85 and F211, a metabolic analysis was performed on watermelon seeds at 13 DAP. The results revealed a total of 32 upregulated and 19 downregulated compounds between BW85 and F211 at 13 DAP (**Supplementary Table S1**). A KEGG classification analysis illustrated that a majority of these compounds belong to the metabolism clade (**Figure 2A**). Furthermore, a comprehensive KEGG analysis uncovered enrichment in 40 pathways (**Supplementary Table S2**). To highlight the most significant pathways, the top 20 pathways were selected based on reliable enrichment (**Figure 2B**). Among the top pathways exhibiting the highest enrichment were biosynthesis of secondary metabolites, ABC transporters, phenylpropanoid biosynthesis, biosynthesis of amino acids, and ubiquinone and other terpenoid-quinone biosynthesis (**Figure 2B**; **Supplementary Table S3**).

### 2.3 Differently expressed genes between BW85 and F211 enriched at ubiquinone and other terpenoid-quinone biosynthesis pathways

**Figure 3:**
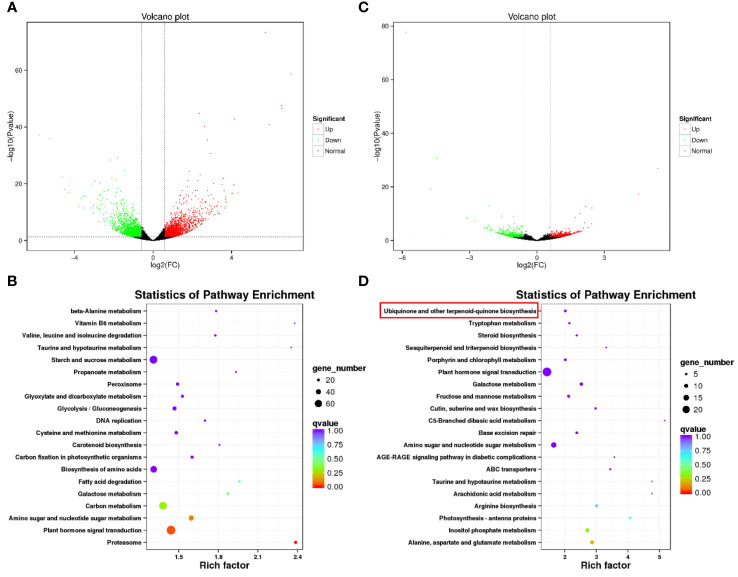
Transcriptomic analysis of seed development between BW85 and F211 at different days after pollination (DAP). **(A)** Volcano plot depicting the transcriptome differences between BW85 and F211 seeds at 13 DAP. **(B)** KEGG enrichment analysis highlighting pathways associated with differentially expressed genes (DEGs) between BW85 and F211 seeds at 13 DAP. **(C)** Volcano plot illustrating the transcriptome variance between BW85 and F211 seeds at 17 DAP. **(D)** KEGG enrichment analysis revealing pathways linked to DEGs between BW85 and F211 seeds at 17 DAP. *n* = 3.

Following the observation of distinct metabolic pathways enriched between BW85 and F211, we aimed to investigate their associated regulatory mechanisms. To accomplish this, a transcriptome analysis was performed for watermelon seeds at 13 and 17 DAP for BW85 and F211, respectively. The analysis at 13 DAP revealed a substantial expression difference, with 1,909 upregulated and 1,839 downregulated genes between BW85 and F211 (**Figure 3A**; **Supplementary Table S4**). The KEGG analysis of the differentially expressed genes (DEGs) unraveled enrichment in 121 pathways (**Supplementary Table S5**). The top pathways with the highest enrichment included the following KEGG pathways: plant hormone signal transduction (73 DEGs), starch and sucrose metabolism (65 DEGs), carbon metabolism (64 DEGs), biosynthesis of amino acids (54 DEGs), and ribosome (46 DEGs) (**Figure 3B**). At 17 DAP, a total of 646 upregulated and 687 downregulated genes were identified between BW85 and F211 (**Figure 3C**; **Supplementary Table S6**). The subsequent KEGG pathway highlighted enrichment in 108 pathways (**Supplementary Table S7**). The top pathways with the highest enrichment included KEGG pathways: plant hormone signal transduction, starch and sucrose metabolism, biosynthesis of amino acids, amino sugar and nucleotide sugar metabolism, and carbon metabolism (**Figure 3D**). These pathways featured 24, 21, 14, 14, and 12 DEGs, respectively. Interestingly, the ubiquinone and other terpenoid-quinone biosynthesis pathways exhibited a significant enrichment, consistent with the results from the metabolic analysis.

**Figure 4:**
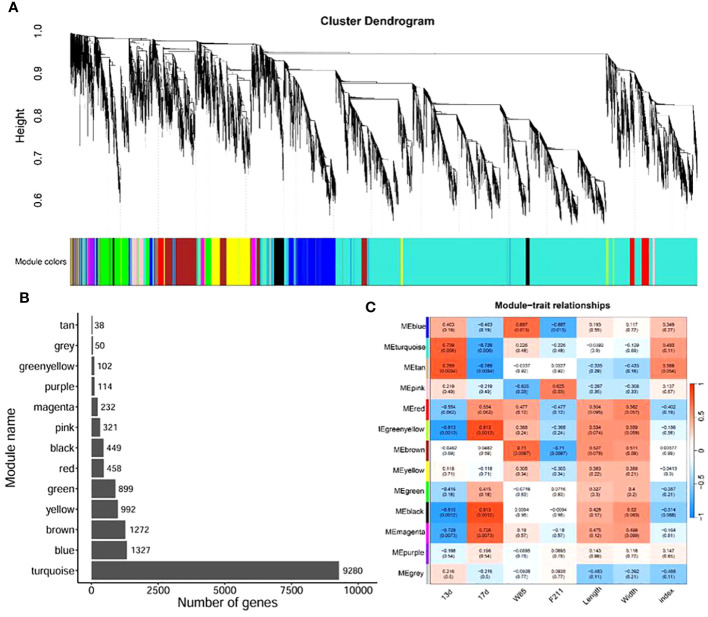
WGCNA of seed development between BW85 and F211 at different DAP. **(A)** Cluster dendrogram showing the different modules of co-expressed genes. The gray module included genes that did not belong to any other modules. **(B)** Distribution of the number of genes contained in different modules. **(C)** Correlation between different modules and sample trait information. The numbers in brackets indicate the *p*-value.

To elucidate the relationship between gene expression patterns and traits, we performed a weighted gene co-expression network analysis (WGCNA). By constructing an unsigned network based on gene expression, we divided them into 13 modules (**Figure 4A**). The module turquoise contains the largest number of genes, followed by modules blue, brown, and yellow (**Figure 4B**). Subsequently, we correlated these modules with the traits of the samples. The results showed that the modules turquoise, tan, green, yellow, black, and magenta were significantly correlated with the development of seeds over time (*p* < 0.05). The modules blue, pink, and brown were significantly related to seed variety. However, there was no significant correlation between modules and seed length, width, and the ratio of the two (**Figure 4C**).

### 2.4 Genetic mapping of seed size in watermelon

**Figure 5:**
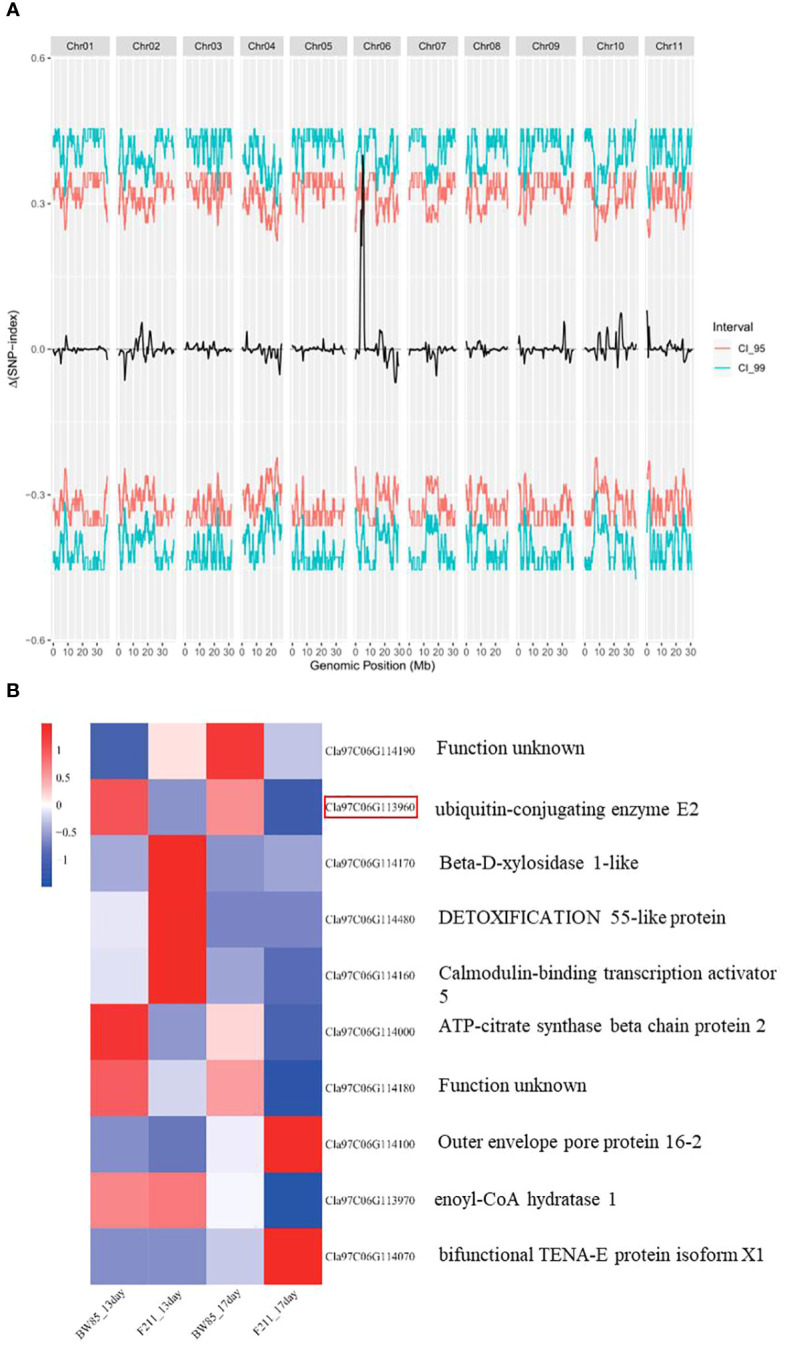
QTL mapping of seed size (SS). **(A)** Distribution of ΔSNP-index in bulked segregant analysis (BSA) for big and small seed sizes. **(B)** Heatmap of differentially expressed genes (DEGs) in the QTL region between BW85 and F211 at 13 and 17 days after pollination.

To genetically map this gene, we conducted a BSA-seq using a population of 200 F_2_ progeny resulting from the cross between BW85 and F211. This mapping identified two adjacent QTLs, designated as *SS6.1* and *SS6.2*, located on chromosome 6 (**Supplementary Table S8**; **Figure 5A**). These QTLs collectively contain 34 annotated genes, according to the watermelon 97103 reference genome sequence (v2.5). More precisely, *SS6.1* extends from Chr06:4847169 to Chr06:5163486, encompassing 33 annotated genes, while *SS6.2* is confined to the genomic interval Chr06:5379337 to Chr06:5419136, which only contains one annotated gene (**Supplementary Table S9**).

Building upon the insights gained from our transcriptomic analysis, we focused our attention on the differentially expressed genes (DEGs) within the QTL region. A total of 10 significant DEGs between BW85 and F211 were identified in the reference watermelon 97103 genome v2.5 (**Figure 5B**; **Supplementary Table S10**). Four of these DEGs appear to be particularly relevant to seed size regulation, with two genes belonging to module blue regulating it in the variety dimension, while two genes belonging to module turquoise play a role in seed growth time. In module blue, Cla97C06G113960, annotated as a ubiquitin-conjugating enzyme E2, is implicated in the ubiquitin pathway that mediated the control of seed size (Li and Li, 2014). Cla97C06G114000, an ATP-citrate synthase beta chain protein 2, is required for normal growth, development, and elongation of C18 to either C20 or C24 fatty acids in seeds (www.mybiosource.com/recombinant-protein/atp-citrate-synthase-beta-chain-protein-2-aclb-2/1461067). In module turquoise, Cla97C06G114480 is a MATE efflux family protein, which is known for its involvement in several physiological functions of plant growth and development (Du et al., 2021). Cla97C06G114100, encoding an outer envelope protein 16, impacts metabolic fluxes during ABA-controlled seed development (Pudelski et al., 2012). Other differentially expressed proteins in this region include an uncharacterized protein (Cla97C06G114190), a beta-D-xylosidase 1-like protein (Cla97C06G114170), a calmodulin-binding transcription activator with an unknown function (Cla97C06G114160), a cAMP-regulated phosphoprotein with an unknown function (Cla97C06G114180), enoyl-CoA hydratase (Cla97C06G113970), and bifunctional TENA-E protein isoform X1 (Cla97C06G114070). Importantly, among all of them, *Cla97C06G113960*, *Cla97C06G114180*, and *Cla97C06G114000* are consistently downregulated at both 13 and 17 DAP (**Figure 5B**).

### 2.5 Comparative genomic analysis of candidate genes in the QTL region

Through a comparative analysis of the genomic sequence from BW85 and F211, a total of 352 variants, comprising 238 SNPs and 114 InDels, were identified within the QTL region (**Supplementary Table S11**). However, all InDels and most of the SNPs were located in the noncoding or intronic regions. Among the 34 candidate genes, only three (*Cla97C06G114010*, *Cla97C06G114030*, and *Cla97C06G114120*) presented non-synonymous (nsSNP) variants between BW85 and F211 (**Supplementary Table S11**). Interestingly, both *Cla97C06G114010* and *Cla97C06G114030* encode pectinesterase inhibitors, each harboring a distinct nsSNP (nucleotide-C68T/protein-S23F and Nucleotide-C305T/Protein-S102F, respectively). Similarly, Cla97C06G114120, presents a single nsSNP (Nucleotide-C977A/Protein-T326N).

## 3 Discussion

Seed size stands as a crucial agronomic trait, impacting the domestication and breeding of plants (Guo et al., 2020). Based on the size, the watermelon seed is classified into six representative types ranging from giant to tomato seed (Kim et al., 2009). Seed size is a complex trait controlled by multiple genes. Historically, Poole and colleagues (1937) identified genes (*l*, *s*, *Ti*, and *ts*) governing seed sizes, while subsequent studies pinpointed approximately 15 genes associated with seed size have been reported to date and major players on chromosomes 2 and 6 (Porter, 1937; Zhang, 1996; Kim et al., 2009; Prothro et al., 2012; Guo et al., 2020). In the present study, we confirmed a major QTL for seed size on LG6 by BSA-Seq analysis of an F_2_ population, from a small and medium size line cross. Moreover, our QTL region revealed new candidate genes related to seed size.

Among the 34 annotated genes in the mapping interval, 10 genes were found to be differentially expressed between BW85 and F211. Notably, *Cla97C06G113960*, *Cla97C06G114180*, and *Cla97C06G114000* were consistently downregulated at both 13 and 17 DAP (**Figure 5B**). Another interesting candidate gene that appeared from our study is Cla97C06G113960, annotated as a ubiquitin-conjugating enzyme E2. Various components of the ubiquitin–proteasome system play important roles in regulating seed development. These components target specific proteins for ubiquitination, marking them for degradation by the proteasome. The ubiquitination process involves the sequential action of three enzymes: ubiquitin-activating enzyme (E1), ubiquitin-conjugating enzyme (E2), and ubiquitin ligase (E3). E1 activates ubiquitin, transferring it to E2, which collaborates with E3 to specify the substrate (Hershko and Ciechanover, 1998; Moon et al., 2004; Zhang et al., 2023). The involvement of the ubiquitin system in determining seed size was noted when a quantitative trait locus controlling grain size and weight in rice was characterized and found to encode the RING-type E3 ligase GW2 (Linden and Callis, 2020). In *Setaria italica*, a RING-type E3 ligase SGD1 and its E2 partner SiUBC32 are involved in regulating grain weight, grain size, and panicle size (Tang et al., 2023). SMALL GRAIN 3 (SMG3) and DECREASED GRAIN SIZE 1 (DGS1), an ERAD-related E2–E3 enzyme pair, regulate grain size and weight through the brassinosteroid (BR) signaling pathway in rice (Li et al., 2023). While E3 ubiquitin ligases, like GW2 and DA1, have been extensively studied for their role in seed size regulation, the functions of E1 and E2 remain less explored (Li and Li, 2014).

Our study identified the ubiquitin-conjugating enzyme E2 gene Cla97C06G113960 as a promising candidate, contributing to the expansion of understanding the less-explored components in ubiquitin-mediated seed size control. While direct evidence linking ubiquitin-conjugating enzymes to the biosynthesis of ubiquinone and terpenoid-quinones may be limited, there is potential for indirect regulation through their involvement in cellular processes such as protein turnover, signaling pathways, and metabolic regulation. Furthermore, scrutinizing the non-synonymous SNP variants among the candidate genes, Cla97C06G114010 and Cla97C06G114030 emerged as noteworthy (**Supplementary Table S11**). Recent studies reported that pectin functions in seed development (Levesque-Tremblay et al., 2015; Xu et al., 2022). Therefore, encoded as pectin esterase inhibitors, Cla97C06G114010 and Cla97C06G114030 have the potential regulatory role in seed size.

In conclusion, our findings expand the understanding of genetic control mechanisms underlying watermelon seed size. The identification of novel genes and the unraveling of their differential expression, coupled with insights into non-synonymous SNPs, open avenues for further research. These insights have implications for advancing plant breeding strategies, facilitating the cultivation of watermelon varieties with optimized seed sizes for various agronomic needs.

## 4 Materials and methods

### 4.1 Plant materials

The F_2_ generation was obtained through the artificial pollination of flowers from the two distinct parental lines, BW85 and F211, which were also used for transcriptome analysis. Notably, BW85 has a larger seed size compared to F211. To ensure controlled conditions, each plant was limited to setting only one fruit.

### 4.2 Mapping strategy

Young leaves from the mapping population were meticulously collected and stored at -80°C. Genomic DNA was extracted from young leaves of watermelon plants by an improved CTAB method (Liu et al., 2022). SNP markers were developed from the whole-genome sequencing data of BW85 and F211, employing a previously described method (Liu et al., 2019). To identify candidate regions associated with each target trait, all of the SNPs between the test samples were summarized based on the alignment results against the reference genome. Subsequently, the Euclidean distance (ED) (Fekih et al., 2013) and Δindex (Hill et al., 2013; Takagi et al., 2013) were calculated. The final QTLs were determined by the intersection of linked regions defined through both ED and Δindex, in conjunction with SNPs.

### 4.3 RNA-seq analysis

RNA extraction was performed on watermelon seeds harvested at 13 and 17 DAP. The Agilent 2100 Bioanalyzer system (Agilent Technologies, CA, USA) was employed to assess RNA quality, ensuring a RIN/RQ (RNA integrity number/RNA quality index)≥8. Sequencing libraries were prepared and sequenced on a HiSeq 4000 platform (Illumina, San Diego, CA, USA), and analysis followed a previously described method (Liu et al., 2021). Genes with thresholds of fold change≥1.5 and *p*-value <0.05 are defined as differentially expressed genes (DEGs). After the logarithmic conversion of FPKM values with a base of 2, the genes with a median absolute deviation (MAD) in the top 75% and greater than 0.01 were used for network analysis. Co-expression gene network analysis was performed using the WGCNA software package v1.72.5 (https://CRAN.R-project.org/package=WGCNA).

### 4.4 Candidate gene prediction and variant calling

To genetically map this gene, we conducted a BSA-seq using a population of 200 F_2_ progeny resulting from the cross between BW85 × F211. This mapping identified two adjacent QTLs, designated as *SS6.1* and *SS6.2*, located on chromosome 6 (**Supplementary Table S1**; **Figure 5A**). These QTLs collectively contain 34 annotated genes, according to the watermelon 97103 reference genome sequence (v2.5). More precisely, *SS6.1* extends from Chr06:4847169 to Chr06:5163486, encompassing 33 annotated genes, while *SS6.2* is confined to the genomic interval Chr06:5379337 to Chr06:5419136, which only contains one annotated gene (**Supplementary Table S2**).

Candidate gene prediction was performed by utilizing the watermelon (97103) v2.5 genome (http://cucurbitgenomics.org/v2/organism/16). The functional annotation of the predicted candidate genes was accomplished using the Dicots PLAZA 4.5 Database (https://bioinformatics.psb.ugent.be/plaza/versions/plaza_v4_5_dicots/) and the BLASTP tool from NCBI (http://blast.ncbi.nlm.nih.gov/Blast.cgi). In addition, variations within the fine mapping interval were identified by analyzing the whole-genome resequencing data of BW85 and F211.

### 4.5 Metabolite analyses

Metabolite extraction was performed on watermelon seeds harvested at 13 DAP. For sample preparation and extraction of widely targeted metabolomics, vacuum freeze-drying was used; the samples were freeze-dried by using a vacuum freeze-dryer (Scientz-100F). The freeze-dried sample was crushed using a mixer mill (MM 400, Retsch) with a zirconia bead for 1.5 min at 30 Hz. Then, 100 mg of lyophilized powder was dissolved with 1.2 mL 70% methanol solution and vortexed for 30 s every 30 min for six times in total. The sample was placed in a refrigerator at 4°C overnight. Following centrifugation at 12,000 rpm for 10 min, the extracts were filtrated (SCAA-104, 0.22 μm pore size; ANPEL, Shanghai, China, http://www.anpel.com.cn/) before UPLC–MS/MS analysis.

The extracts were analyzed using an UPLC–ESI–MS/MS system (UPLC, SHIMADZU Nexera X2, www.shimadzu.com.cn/; MS, Applied Biosystems 4500 Q TRAP, www.appliedbiosystems.com.cn/). The analytical conditions were as follows: UPLC: column, Agilent SB-C18 (1.8 µm, 2.1 mm × 100 mm). The mobile phase consisted of solvent A, pure water with 0.1% formic acid, and solvent B, acetonitrile with 0.1% formic acid. Sample measurements were performed with a gradient program that employed the starting conditions of 95% A, 5% B. Within 9 min, a linear gradient to 5% A, 95% B was programmed, and a composition of 5% A, 95% B was kept for 1 min. Subsequently, a composition of 95% A, 5.0% B was adjusted within 1.10 min and kept for 2.9 min. The flow velocity was set as 0.35 mL per minute. The column oven was set to 40°C. The injection volume was 4 μL. The effluent was alternatively connected to an ESI-triple quadrupole-linear ion trap (QTRAP)-MS.

For ESI-Q TRAP-MS/MS analysis, the ESI source operation parameters were as follows: source temperature, 550°C; ion spray voltage (IS), 5,500 V (positive ion mode)/–4,500 V (negative ion mode); ion source gas I (GSI), 50 psi; gas II (GSII), 60 psi; curtain gas (CUR), 25 psi. The collision-activated dissociation (CAD) was high. QQQ scans were acquired as MRM experiments with collision gas (nitrogen) set to medium. DP (declustering potential) and CE (collision energy) for individual MRM transitions were done with further DP and CE optimization. A specific set of MRM transitions was monitored for each period according to the metabolites eluted within this period.

Both hierarchical cluster analysis (HCA) and Pearson’s correlation coefficient (PCC) analysis of the samples and metabolites were carried out using the ComplexHeatmap package in R. Variable importance in projection (VIP) scores >1 and |log_2_FC| ≥1.0 were used to determine differentially abundant metabolites. The VIP values were extracted from the result of the orthogonal projections to latent structures discriminant analysis (OPLS-DA).

## 5 Conclusion

In summary, our study has successfully identified a major QTL on chromosome 6 that serves as a regulator of watermelon seed size. Within the designated mapping interval, we have cataloged a total of 34 annotated candidate genes. Particularly noteworthy are three promising candidates—Cla97C06G113960 (ubiquitin-conjugating enzyme E2), *Cla97C06G114010*, and *Cla97C06G114030* (pectin esterase inhibitors)—which emerged from a comprehensive transcriptomic and genomic analysis. Future research should focus on the functional verification of these candidates, which opens a valuable opportunity for the development of watermelon varieties with customized seed sizes, catering to diverse consumer preferences and market demands.

## Data availability statement

The datasets presented in this study can be found in online repositories. The names of the repository/repositories and accession number(s) can be found in the article/**Supplementary Material**.

## Author contributions

XW: Conceptualization, Data curation, Funding acquisition, Investigation, Methodology, Writing – original draft, Writing – review & editing. WY: Conceptualization, Investigation, Methodology, Software, Writing – review & editing. NR: Writing – review & editing. YJ: Resources, Writing – review & editing. YF: Data curation, Writing – review & editing. XZ: Funding acquisition, Supervision, Writing – review & editing. HY: Supervision, Writing – review & editing. YC: Supervision, Writing – review & editing. BL: Conceptualization, Funding acquisition, Software, Writing – review & editing.

## Supplementary material

Supplementary Table 1 Different metabolic compounds between BW85 and F211 at 13 DAP.

Supplementary Table 2 KEGG classification annotation of different metabolic compounds between BW85 and F211 at 13 DAP.

Supplementary Table 3 KEGG pathways enrichment analysis of different metabolic compounds between BW85 and F211 at 13 DAP.

Supplementary Table 4 Differentially expressed genes (DEGs) between BW85 and F211 at 13 DAP.

Supplementary Table 5 KEGG annotation of DEGs between BW85 and F211 at 13 DAP.

Supplementary Table 6 Differentially expressed genes (DEGs) between BW85 and F211 at 17 DAP.

Supplementary Table 7 KEGG annotation of DEGs between BW85 and F211 at 17 DAP.

Supplementary Table 8 QTLs for watermelon seed size.

Supplementary Table 9 Annotation of genes in the QTL region.

Supplementary Table 10 DEGs in the QTL region.

Supplementary Table 11 InDels and SNP annotation in the QTL region.
